# Factors affecting effective ventilation during newborn resuscitation: a qualitative study among midwives in rural Tanzania

**DOI:** 10.1080/16549716.2018.1423862

**Published:** 2018-01-18

**Authors:** R. Moshiro, H. L. Ersdal, P. Mdoe, H. L. Kidanto, C. Mbekenga

**Affiliations:** ^a^ Department of Paediarics and Child Health, Muhimbili National Hospital, Dar es Salaam, Tanzania; ^b^ Department of Health Studies, University of Stavanger, Stavanger, Norway; ^c^ Department of Anesthesiology and Intensive Care, Stavanger University Hospital, Stavanger, Norway; ^d^ Department of Obstetrics and Gynecology, Haydom Lutheran Hospital, Manyara, Tanzania; ^e^ Ministry of Health Community Development, Gender, Elderly and Children, RMNCH Section, Dar es Salaam, Tanzania; ^f^ Department of Research, Stavanger University Hospital, Stavanger, Norway; ^g^ School of Nursing and Midwifery, Aga Khan University, Dar es Salaam, Tanzania

**Keywords:** Helping Babies Breathe, barriers bag mask ventilation, facilitators bag mask ventilation, simulation training, qualitative Tanzania

## Abstract

**Background:** Intrapartum-related hypoxia accounts for 30% of neonatal deaths in Tanzania. This has led to the introduction and scaling-up of the Helping Babies Breathe (HBB) programme, which is a simulation-based learning programme in newborn resuscitation skills. Studies have documented ineffective ventilation of non-breathing newborns and the inability to follow the HBB algorithm among providers.

**Objective:** This study aimed at exploring barriers and facilitators to effective bag mask ventilation, an essential component of the HBB algorithm, during actual newborn resuscitation in rural Tanzania.

**Methods:** Eight midwives, each with more than one year’s working experience in the labour ward, were interviewed individually at Haydom Lutheran Hospital, Tanzania. The audio recordings were transcribed and translated into English and analysed using qualitative content analysis.

**Results:** Midwives reported the ability to monitor labour properly, preparing resuscitation equipment before delivery, teamwork and frequent ventilation training as the most effective factors in improving actual ventilation practices and promoting the survival of newborns. They thought that their anxiety and fear due to stress of ventilating a non-breathing baby often led to poor resuscitation performance. Additionally, they experienced difficulties assessing the baby’s condition and providing appropriate clinical responses to initial interventions at birth; hence, further necessary actions and timely initiation of ventilation were delayed.

**Conclusions:** Efforts should be focused on improving labour monitoring, birth preparedness and accurate assessment immediately after birth, to decrease intrapartum-related hypoxia. Midwives should be well prepared to treat a non-breathing baby through high-quality and frequent simulation training with an emphasis on teamwork training.

## Background

Of the 130 million babies born each year worldwide, three million will die within the first four weeks of life. The burden of neonatal mortality rests almost entirely on poor countries with an estimated one million newborn deaths occurring in Sub-Saharan Africa each year. Intrapartum-related hypoxia accounts for more than a quarter of these deaths, and contributes to an unknown number of disabilities [1,2].

The goal of early basic resuscitation of an apneic newborn is reversal of the hypoxic-ischemic process and, ultimately, initiation of spontaneous respirations. Accurate evaluation of heart rate and respiration, coupled with prompt initiation of basic resuscitation interventions is thought to be critical for successful neonatal outcome [3]. In 2009, the Tanzanian Ministry of Health, Community Development, Gender, Elderly and Children introduced a learning programme called Helping Babies Breathe (HBB) [4], to teach midwives basic newborn resuscitation to help reduce the burden of perinatal hypoxia. The initial evaluation, conducted at eight sites, showed a 47% reduction in early neonatal deaths within 24 hours, and a 24% reduction in fresh stillbirths [5]. Furthermore, the roll-out of HBB has helped to supply health facilities with important resuscitation equipment [6]. Haydom Lutheran Hospital (HLH) was one of the initial eight HBB sites, and several quantitative studies related to HBB have been conducted there [5,7–9]. In 2013, building on the HBB programme, a large research and innovation project named Safer Births started, and all delivery rooms at HLH were equipped with newborn resuscitation monitors (Laerdal Global Health). These monitors record various ventilation parameters, such as heartrate, expired volume, expired CO_2_, and inspiratory pressure for research purposes; however, only heart rate is displayed on the monitor visible to the provider during resuscitation [10,11].

At HLH, frequent HBB simulation training was shown to improve clinical outcomes, indicating a successful transfer of new knowledge and skills into clinical practice [7]. However, several studies have identified problems during actual newborn resuscitation, including the inability to quickly identify a newborn who needs assistance, the inability to correctly identify and use the heart rate to guide decisions, delays in initiating BMV, and the inability to administer effective ventilation [8,12–14]. These studies recommended more training and research to increase insight on how to improve newborn resuscitation.

Alternatively, few qualitative studies have investigated the midwives’ perspective on the challenges related to newborn resuscitation in low-income countries. A lack of equipment, inadequate skills and knowledge, and ineffective communication are among the factors reported to hinder effective resuscitation, while training and good education are thought to decrease newborn morbidity and mortality [15,16]. Because low-income countries contribute significantly to the burden of intrapartum asphyxia, there is a need to better understand the context-specific factors that affect effective ventilation within these regions.

The aim of this study was to explore factors affecting effective ventilation during newborn resuscitation. Specifically, the barriers and facilitators to BMV and midwives’ experiences of using the newborn resuscitation monitor were explored. Because newborn ventilation is mainly performed by midwives in Tanzania, their thoughts, views and experiences will offer great insight on how ventilation practices can be improved in the future.

## Methods

### Study area and context

HLH is situated in Mbulu District of Manyara Region, northern Tanzania. The immediate catchment area covers a population of approximately 450,000. HLH has approximately 5000 deliveries annually, and midwives largely conduct all deliveries. Doctors do not attend all deliveries; however, they are on call 24 hours a day to attend emergencies, including caesarean section, when called upon. On admission, women are assessed by the intern doctors on call and a midwife. For high-risk pregnancies, and other obstetric complications during labour, an obstetrician is called to review the patient. Otherwise, midwives will continue with labour management using partograph until the baby is delivered. Basic resuscitation of the newborn is performed by the attending midwives with the help of nurse assistants. Due to the high turnover of hospital staff, not all midwives have undergone full HBB training; some have only participated in short resuscitation training sessions. All nurses who were interviewed held a diploma in nursing, which is a three-year training programme to become a registered nurse midwife (RNM).

### Study design

This was a qualitative study where individual, semi-structured interviews with midwives were conducted and audio recorded using a digital audio recorder. The individual interview method was chosen because it provides insight into people’s thoughts, feelings and behaviours on important and lifesaving issues such as resuscitation [17].

### Sampling and sample size

Midwives who had worked in the labour ward for more than one year and who had conducted more than three newborn resuscitations during that time were eligible for selection. This ensured the inclusion of information-rich informants [18]. Participants were recruited until a saturation point was reached, where no more new information was extracted from the data [17,19,20].

### Data collection

Interview sessions were conducted within the hospital premises, in a private office at the convenience of the participant. The first author conducted two initial interviews to finalise the interview guide. The interview guide had questions under the following headings: communication and cooperation during resuscitation; initiation of ventilation; continuation of ventilation; resuscitation training; resuscitation protocol; and resuscitation monitoring.

The rest of the interviews were conducted by one research assistant: a psychologist working at the hospital. He was introduced to the interview guide and then he conducted one pilot interview, which was approved by the rest of the research group. The interviews lasted an average of 40–50 minutes.

At the end of each interview day, RM together with one senior researcher in our group, reviewed the information that had been collected to generate an emerging understanding regarding newborn ventilation before the subsequent interview session. This iterative process of data collection and review was used to shape the questions being asked in the subsequent interviews, as well as to recognize the point at which no new information emerged [21]. The interviews were conducted in Swahili.

### Analysis

The audio recordings were transcribed and then translated into English for our English-speaking researchers. Each paragraph was translated and positioned below the original Swahili paragraph to preserve the meaning of the whole paragraph. The first author checked each transcription and translation to ensure its quality, and minor mistakes were corrected before analysis began. The interview transcripts were then read and re-read to identify text relevant to newborn ventilation. Qualitative content analysis [22] was preferred as the method of analysis because it allows for data to be interpreted and acted on for their meanings. Using this method, the text containing key thoughts about ventilation was then condensed, abstracted and labelled with a code. The codes were re-visited to remove text that was not relevant to our research question but also to avoid repetitions. We discussed the codes and grouped similar codes to obtain sub-categories, and similar sub-categories were grouped into categories (Table 1). RM, CKM and other members of the research team were responsible for sorting and obtaining categories using a constant comparative method [21]. NVivo 11 software was used to facilitate the generation and sorting of codes.

**Table 1.** Illustration of how codes, sub-categories and categories were obtained.

Interview quote	Code	Sub-category	Category
What helps is the initial preparation, before any delivery we prepare our equipment making sure they function well. (RNM 2)	Preparation of equipment’s before delivery	Preparation of equipment and staff	
For now it is not difficult since we have it in place, not like in the past where when the baby is born that’s when you run to look for it ‘guys can I have a mask, can I have this’ (RNM 1)	Resuscitation equipment located in one place		
Sometimes it happens that you deliver another woman in the labour room that it’s equipment has already been used without cleaning, it leads to delays in helping another baby (RNM 5)	Uncleaned equipment leads to delays starting ventilation		Proper monitoring of labour and preparation for delivery helps to start ventilation on time
First thing is to detect a baby who will need resuscitation during monitoring of labour, that the woman is going to deliver soon and there is abnormal foetal heart rate (RNM 2)	Monitoring of foetal heart rate during labour	Close monitoring of labour to detect problems early	
You will have doubts since she had prolonged second stage, and you wonder if the baby will come out OK, you will have to call for help because you don’t know what will happen after delivery (RNM 3)	Prepared to do resuscitation after diagnosing foetal distress		

## Results

Eight midwives, aged between 26 and 47 years with a median of 8 years of experience in the labour ward, were interviewed between December and January 2016. Each had performed at least 10 resuscitations in her career. The main categories identified that influence effective ventilation were: 1) proper monitoring of labour and preparation for delivery helps to start ventilation on time; 2) teamwork among midwives during resuscitation determines how quickly ventilation could be initiated; 3) increased frequency of training with improved manikins improved newborn ventilation; 4) challenges in assessing clinical responses delay the next course of action; and 5) anxiety and fear affect technique and performance of ventilation (Table 2).

**Table 2.** Categories and sub-categories in order of their importance according to how many times it was mention by the midwives to affect ventilation.

Category in order of importance as reported by midwives	*Sub-category*
1. Increased frequency of training to improve newborn ventilation	*1. Increased frequency of self-practice to improve knowledge, skills and confidence during ventilation*
	*2. Improving simulation training to prepare midwives for ventilation*
2. Monitoring of labour and preparation for delivery help to start ventilation on time	*1. Preparation of equipment*
	*2. Labour monitoring*
3. Teamwork and commitment during resuscitation determine how quick ventilation can be initiated	*1. Cooperation*
	*2. Non-caring attitude*
4. Difficulties interpreting clinical responses delay subsequent actions during resuscitation	*Resuscitation monitor provides quick assessment without interrupting ventilation*
5. Anxiety and fear affect technique and ventilation performance	

### Monitoring of labour and preparation for delivery help to start ventilation on time

#### Labour monitoring

Midwives mentioned the importance of close monitoring of labour for early detection of problems and preparedness for starting timely when necessary. They mentioned the importance of being aware of the possibilities of delivering a depressed baby in need of help. Good monitoring of labour was reported to be associated with a decreased need for resuscitation, but, if indicated, the health-care workers would be ready:

Once we see a woman has been labouring for a long time, we get prepared to resuscitate the baby because we know anything can happen. If we are three of us, the one conducting delivery will continue to do so, another one will wear gloves and wait for the baby while the third one will be around ready to assist. This is when we see indications that the baby is tired. (RNM 4)

#### Preparation of equipment

Midwives reported that the preparation of equipment, and sometimes staff, for unforeseeable and foreseeable resuscitations, helps them to start ventilation on time, reduces anxiety and increases the chances of a baby surviving resuscitation. They reported the importance of storing equipment in a dedicated place and inspecting it before each delivery, making it readily available when needed. This practice was adopted during the implementation of the monitors that include the bag and mask. These are mounted on the wall in front of the resuscitation table and it is easy to see if the bag is missing. Every morning, one dedicated person inspects whether the monitors and bag-mask are in place and functioning.

I get prepared once I suspect that the newborn will not breathe, therefore, I check my equipment, and if they are not well prepared, I will prepare them for resuscitation. (RNM 5)

Poor preparation or misplaced equipment was said to lead to delays in starting ventilation, by wasting time having to look for it. Midwives reported a situation where some of the used equipment, such as bags and masks, were found to be dirty at a time when they were needed urgently.

### Teamwork and commitment during resuscitation determine how quickly ventilation could be initiated

#### Cooperation

According to the midwives, resuscitation is performed more effectively by two or more midwives helping each other. One midwife will ventilate the baby, while the others will be watching and monitoring the actions of the rescuer. Although the number of midwives at the hospital does not allow them to work in pairs at all times, they do call each other for assistance prior to delivery. Being with a colleague during delivery was reported to be important even if there were no anticipated problems:

I normally don’t allow myself to deliver a woman when I am alone, I will always call my colleague to be around no matter how busy they are, even if I see no indications that I will encounter problems, because you never know what will happen. (RNM 4)

Working together as a team enables midwives to use each other’s strengths when they are faced with difficulties. In the presence of colleagues, midwives can communicate ways of improving on-going resuscitation, such as covering the baby to maintain warmth or alerting the person ventilating to adjust the face-mask to obtain a better mask seal should they note poor chest-rise. In other circumstances, midwives who are observing the resuscitation might take over the ventilation if they think their colleague is struggling to ventilate. One midwife commented;

That is a possibility [taking over ventilation], once you see that she is not managing to get air in, then you ask to help her, and what we care about is the life of the baby, therefore you can’t keep quiet, you ask her to let another person ventilate. (RNM 3)

Alternatively, midwives shared their experiences of not being organized, leading to confusion, interference and interruptions in ventilation when resuscitation is attended by more than two midwives. They reported that it is not routine to agree on the role for each midwife during resuscitation, especially when resuscitation was not anticipated. However, one midwife will always be prepared to receive the baby after the cord has been separated.

Sometimes I might cover the baby but left the chest uncovered so that I see if I am getting air in, but another person will only think that I am exposing the baby therefore will come and cover the baby. Sometimes when there are many of us it is not good. (RNM 6)

#### Non-caring attitude

It was reported by the midwives that a few of their colleagues had questionable attitude and drive when saving lives during labour and delivery because of the way they respond to emergencies. This, in turn, can slow down their response to a non-breathing baby when a colleague is in need of help and hence delay the initiation of ventilation:

There is this attitude or behaviour of not caring, you might find your colleague has no patient in her delivery room and you happen to have an emergency like you have delivered an asphyxiated baby in your room, you call her to come and help, and she comes very slowly, like it’s not an emergency or like she doesn’t want to help. (RNM 2)

When one midwife was questioned about whether she or other health care workers have tried to confront midwives with such attitude, she responded that they do not take any action in order to maintain a social relationship, especially outside the working environment.

#### Lack of joint decision-making between midwives and doctors

It was further reported that sometimes the opinions of midwives are not taken into consideration when doctors make decisions about women in labour. This lack of collaboration between doctors and midwives stems from the fact that doctors have the final decision, and midwives are left without an option, only to document what they have done or what they have been advised to do. Midwives felt that this adds to the burden of babies who need to be ventilated unnecessarily. One midwife recounted a previous event:

I told the doctor that it would be difficult to deliver this baby, but he reviewed and examined the mother, and he said she would deliver. Later I told the doctor you will not leave until we have delivered this baby, and once the baby came out we had to resuscitate for a long time but later the baby died. (RNM 4)

### Increased frequency of training to improve newborn ventilation

#### Increased frequency of self-practice to improve knowledge, skills and confidence during ventilation

Midwives suggested that the only way to improve their performance is through increased training frequency and repeated self-practice to acquire the techniques and skills necessary for performing resuscitation. It was pointed out that the HBB programme helped them to increase their perceived confidence during resuscitation, although it took them some time before they were comfortable with the algorithm:

When you see a baby is flat after initial drying, you start to call for help, then you try to perform suctioning without knowing where to start, from the mouth or nose, sometimes you even cut the cord before stimulation and you start bagging before even suctioning, in-short, we used to panic. But later, when we continued to practice we came to understand step after step, where to start and proceed until the end. (RNM 2)

Midwives agree that ventilation skills take time and practice to be acquired. They also mentioned a variation in levels of knowledge and skills between midwives, which contributes to increased interruptions during ventilation and delays in starting ventilation. Interrupting ventilation was thought to be due to a lack of knowledge about its consequences.

Ventilation practice used to take place twice weekly after the morning report for some time at Haydom Hospital. However, that practice was stopped after the person who initiated it left the hospital. Midwives thought it was a very good idea, as, during the sessions, there would be a supervisor watching them while they practiced, and it was an opportunity for them to be corrected if there was a certain skill they were not doing correctly. There was a suggestion from one midwife that the practical training should be followed by supervision during the actual resuscitation of babies in the labour ward:

Maybe, during training, supervisors should be with us in the labour ward to witness two or three actual resuscitations so that they can continue to correct us whenever we do something wrong, because we will be doing it on real babies and not manikins. (RNM 4)

#### Improving simulation training to prepare midwives for ventilation

Midwives pointed out that the current skills training is not sufficient to prepare the midwives for actual resuscitation in the labour ward. The sense of urgency is missing. In addition, the manikins do not respond in any way when some of the interventions are performed on it:

If there was a way of improving the manikin so that when we are stimulating there is something that shows you some improvement, like right now when you stimulate the manikin, nothing happens. (RNM 2)

### Difficulties interpreting clinical responses delay subsequent actions during resuscitation

Immediately after delivery, midwives are supposed to quickly assess the newborn to determine whether it needs stimulation, suctioning and/or ventilation. Midwives mentioned a number of clinical signs that they look for to determine the next course of action. Such signs include whether the baby is breathing, its colour and activity, as well as its heart rate.

Midwives reported that failure to recognize early signs that a baby needs ventilation leads to a delay in starting ventilation, as they will be busy stimulating or suctioning:

After stimulation and suction, sometimes you can see the baby responding, therefore you wait, but as you continue to wait, you see the colour starts to change and the breathing continues to be abnormal, but you have already wasted some time when you thought the baby would come up. (RNM 1)

#### Resuscitation monitor provides quick assessment without interrupting ventilation

Conversely, midwives reported that the resuscitation monitors help them to quickly determine the newborn’s heart rate once there is a need to start ventilation. The monitor was reported to quickly provide heart rate as feedback during ongoing ventilation, as opposed to interrupting ventilation for auscultation (which was necessary in the past).

But those monitors help, after initial stabilization, instead of using the stethoscope, you just attach the sensor, and you see on the screen the heart rate, therefore it makes things easy. (RNM 5)

Alternatively, midwives reported incidences when the monitor was not functioning in one of the labour rooms, making it necessary to move the baby to another room for resuscitation, which led to the loss of precious time for resuscitation.

### Anxiety and fear affect technique and ventilation performance

Midwives reported that they sometimes became anxious once they realize that a baby is depressed. The fear and/or anxiety was reported to arise because of the pressure they feel to ensure the baby survives. When there is a possibility that the baby might not survive, or when the baby does not respond after a period of ventilation, the fear may turn to panic:

… you become fearful thinking whether you will be able to save that life … that is why sometimes we panic. (RNM 4)

The fear/anxiety was reported to affect the way they ventilate the baby, such as the technique of holding the bag and mask, interrupting ventilation and skipping some steps of the guidelines. Additionally, fear was reported to be responsible for time loss during resuscitation:

Once you have fear, you will not be able to hold it properly (the bag and mask) the way we have been instructed to, and you will get air leaks because you are shaking, you have fear, and the baby will not get the air you are giving it. (RNM 1)

## Discussion

This study highlights the main facilitators and barriers currently facing Tanzanian midwives in their daily practice while attempting to rescue non-breathing babies at birth. Midwives’ teamwork during resuscitation was mentioned as being an important factor in facilitating ventilation. Participants were clearly happy to help each other perform certain tasks once they were summoned. However, there was no pre-arrangement of the roles for each of the team members, leading to confusion or interference, and the exchange of roles during the actual intervention.

The lack of pre-arranged roles stems from the fact that HBB training is tailored to resource-limited settings where, mostly, resuscitation is performed by a single rescuer. Simmons et al. reported the importance of having clear roles and responsibilities during emergency and stressful interventions as being critical for positive outcomes [23]. Jordanian midwives reported lack of teamwork as a barrier to successful neonatal resuscitation [15]. All the nurses had received HBB training, which does not have a component for team-training. We therefore think it is important to consider teamwork during simulation training so that midwives can practise their roles and responsibilities in the delivery room.

A lack of joint decision-making between midwives and doctors has been reported previously in Tanzania [24]. Midwives and doctors frequently disagree on the management of patients, such as the decision to perform caesarean section [24]. In this study, midwives thought that sometimes their opinions were being ignored, despite their perceived clinical experience. Such conflicts might not seem to affect ventilation directly, but may affect team spirit, respect, confidence and communication between providers, and, subsequently, affect care and the outcome of some deliveries. It is therefore important for the management of health facilities to create an environment in which both midwives and doctors can work in harmony and with good understanding. This can be achieved by providing joint clinical obstetric training for both doctors and midwives.

Some midwives were reported as having a non-caring attitude when saving newborns during resuscitation. Although this comment was directed at only a few individuals, other studies in Tanzania and Kenya have highlighted similar issues, such as midwives’ lack of motivation due to poor working conditions, and the feeling of not being recognized by their supervisors [25–28]. Motivating health-care workers in resource-poor settings can sometimes be a challenge. Nevertheless, supportive leadership and effective management at hospital level has been shown to modify the impact of resource shortfalls and foster good working relations between cadres [26,29].

The importance of appropriate labour monitoring, together with the preparation of resuscitation equipment, was mentioned as the most effective way of facilitating resuscitation. The fact that labour monitoring was highlighed as a facilitator of ventilation probably reflects that a sub-standard labour monitoring practice is taking place in this low-resource setting [30–32]. The monitoring of labour is one of the important steps in ensuring good foetal outcome; therefore, efforts should be geared towards improving and standardising labour monitoring for the benefits of the fetus and the mother.

After the introduction of HBB, health-care facilities in Tanzania were equipped with basic resuscitation equipment, therefore, a lack of equipment was not mentioned as a barrier, as has been reported in other low-income countries [15,16]. The World Health Organization (WHO) recommends the preparation of delivery equipment and supplies, including newborn resuscitation equipment, whenever delivery is anticipated [33]. This view was shared by the midwives, as they emphasized the importance of having equipment ready, to be able to initiate ventilation within the first minute. Although the outcomes of resuscitation attempts depend on many things, such as the skillful use of resuscitation equipment, it is still vital to ensure that equipment is ready whenever it is needed.

Previous studies have reported the importance of frequent simulation training, as opposed to a single training session. Single training sessions have been associated with adequate skills retention [9,34]; however, frequent brief on-site sessions were shown to retain skills as well as improve the clinical outcomes of resuscitated babies [6]. Midwives reported that more practical training would increase their knowledge, skills, competence and confidence in performing ventilation. They went further to suggest that offering short training sessions during working hours, as opposed to self-practice alone, would make a difference in terms of outcome. A preference for more realistic simulation training was also brought up during the interviews. All of the midwives in this study had only received basic resuscitation training using the NeoNatalie (Laerdal Global Health) newborn manikin, which is a low-fidelity manikin. Simulation has many advantages, and results in highly trained health-care workers who are less likely to make life-threatening or costly medical errors [35].With adequate training, midwives will be able to respond quickly and efficiently to the needs of asphyxiated babies. Many high-income countries have already established advanced simulation training centres, a resource which also needs to be established in low-income countries. Furthermore, the introduction of debriefings as a learning tool during simulations, as well as after a serious clinical event, will help improve performance. Debriefing is a process in which people who have had a certain experience are led through a purposive discussion regarding the experience [36,37]. Unlike audits, debriefing helps the providers to learn and reflect on what they have experienced immediately after an actual or a simulated event, giving them a chance to learn from their experience.

Although the clinical signs used to assess newborns immediately after delivery are well known (Apgar: colour, tone, heart rate, respirations and reflexes), still there seems to be challenges in assessing a newborn baby correctly, which contributes to delays in instituting treatment or interventions. The Apgar score has been accepted for many years as a standard tool for the assessment of newborns immediately after delivery, despite being subjective and unreliable [38,39]. This study suggests that incorrect assessment and interpretation of the clinical signs are delaying the initiation of ventilation or influencing interruptions during ventilation. One way to improve this practice is through advanced simulation training, where dynamic, complex and unanticipated situations could be practised and managed. Midwives furthermore reported the usefulness of the resuscitation monitor to quickly determine the initial heart rate and heart rate responses to interventions; there is no need to auscultate for heart rate, avoiding unnecessary ventilation pauses. Midwives reported that displaying the time is crucial for them to keep track and see how many minutes have elapsed since resuscitation started. The monitor, when used properly, can help both in the initial assessment of the heart rate and as a feedback mechanism during actual ventilation.

Incidences of fear or anxiety when faced with a baby in need of ventilation came up during our discussions. Fear and anxiety was reported to affect ventilation performance and an inability to follow the HBB protocol during resuscitation. According to the midwives, fear or anxiety was a result of them feeling pressure to ensure that the baby survives. There could be a number of factors causing anxiety in these situations. Health-care workers may be questioned about perinatal deaths occurring during their shifts, especially if the mother had a live fetus during admission. One study, conducted in urban Tanzania, reported that midwives and doctors fear blame from peers or management during perinatal and maternal audits [24]. Furthermore, acute stressful medical situations tend to induce anxiety, which may affect performance, especially when health workers perceive themselves as not having enough resources to respond to the situation [40,41]. At HLH, maternal and perinatal audits do take place and this could be one of the contributing factors to the fear and anxiety. Audits are very important, but they need to be conducted in an appropriate and constructive manner, without naming, blaming or shaming health-care workers. Resuscitation training should have interventions geared towards improving performance under stress, or interventions for midwives to cope with and withstand stressors.

In this study, we ensured credibility by using midwives’ narratives to describe their shared experiences in relation to newborn resuscitation. By using in-depth interviews, we believe we have chosen the best data collection method to address our objectives and confirm our credibility. Participants were selected carefully, considering their years of service as well as the number of resuscitations that they had. We included participants of various ages to increase variation. During analysis, we used qualitative content analysis, which allowed us to examine the text and make sense of it. We also ensured that all relevant data were included in our categories by involving more than one researcher [22].

The use of an interview guide ensured consistency while keeping the sessions open enough for midwives to share their experiences. Initial interviews were used to modify the interview guide, and subsequent interviews took into consideration the information that was already collected, ensuring that we captured changes over time in data collection. Our participants were free to share their experiences during interviews due to the use of Swahili as the medium of communication. Our research group comprised midwives, paediatricians, obstetricians and anaesthesiologists, who brought with them extensive knowledge on newborn resuscitation, which helped to improve confirmability. Providing details about the context in which this study was carried out, as explained in detail in the methods section, together with quoted text in the results section, will help readers to compare these findings with their own context and decide the transferability of our findings.

Among the limitations of this study is our decision not to interview doctors who also work with nurses during labour and delivery. We made this decision because we thought that their contribution to the subject of newborn resuscitation would be limited, as they normally do not participate in resuscitations. Furthermore, we acknowledge the inclusion of midwives from only one rural hospital in Tanzania. Interviewing midwives from another facility in an urban area could have provided more insights and thus enriched our discussion.

### Conclusion and recommendations

We have identified the ability to monitor labour properly, preparing resuscitation equipment before delivery, teamwork and frequent ventilation training as factors that facilitate effective ventilation. Barriers to effective ventilation were mentioned as being anxiety and/or fear during ventilation, and difficulties in assessing clinical responses during ventilation.

To improve the outcomes of resuscitated babies, we need skilled midwives who are competent and comfortable in resuscitation skills, including appropriate assessment of the newborn’s condition immediately after delivery. Continued efforts are needed to improve simulation training through the use of more realistic manikins as well as increased frequency of practice. We have highlighted the importance of joint decision-making between midwives and doctors: it is high time that teamwork organization is introduced during resuscitation training in low-income countries.

Future studies on resuscitation in low-resource countries should focus on the best ways to improve training, including incorporating teamwork training, in an environment with a scarcity of health-care workers.

## Paper context

Improving newborn resuscitation in low-income countries is critical to newborn survival. This study explored the views of midwives on how to improve newborn resuscitation. Midwives reported simple measures such as simulation training, preparation of equipment pre-delivery, labour monitoring and accurate assessment at birth to be equally important for resuscitation outcome. Fear, anxiety and the lack of team-training led to confusion and misunderstandings during resuscitation. This implies that resuscitation training should focus more on adequate preparation and team-training.
